# Crimean-Congo hemorrhagic fever cases diagnosed during an outbreak of Sudan virus disease in Uganda, 2022–23

**DOI:** 10.1371/journal.pntd.0012595

**Published:** 2024-10-16

**Authors:** Stephen Balinandi, Sophia Mulei, Shannon Whitmer, Luke Nyakarahuka, Caitlin M. Cossaboom, Elizabeth Shedroff, Maria Morales-Betoulle, Inna Krapiunaya, Alex Tumusiime, Jackson Kyondo, Jimmy Baluku, Dianah Namanya, Calvin R. Torach, Joanita Mutesi, Jocelyn Kiconco, Godfrey Pimundu, Tonny Muyigi, Jessica Rowland, Andrew Nsawotebba, Isaac Ssewanyana, David Muwanguzi, Daniel Kadobera, Julie R. Harris, Alex R. Ario, Kagirita Atek, Henry B. Kyobe, Susan Nabadda, Pontiano Kaleebu, Henry G. Mwebesa, Joel M. Montgomery, Trevor R. Shoemaker, Julius J. Lutwama, John D. Klena

**Affiliations:** 1 Uganda Virus Research Institute, Entebbe, Uganda; 2 Viral Special Pathogens Branch, Centers for Disease Control and Prevention, Atlanta, United States of America; 3 College of Veterinary Medicine, Animal Resources and Biosecurity, Makerere University, Kampala, Uganda; 4 Ministry of Health, Kampala, Uganda; 5 Uganda Public Health Fellowship Program, Kampala, Uganda; 6 Division of Global Health Protection, Centers for Disease Control and Prevention, Atlanta, United States of America; NIAID RML: National Institute of Allergy and Infectious Disease Rocky Mountain Laboratories, UNITED STATES OF AMERICA

## Abstract

**Background:**

In September 2022, Uganda experienced an outbreak of Sudan virus disease (SVD), mainly in central Uganda. As a result of enhanced surveillance activities for Ebola disease, samples from several patients with suspected viral hemorrhagic fever (VHF) were sent to the VHF Program at Uganda Virus Research Institute (UVRI), Entebbe, Uganda, and identified with infections caused by other viral etiologies. Herein, we report the epidemiologic and laboratory findings of Crimean-Congo hemorrhagic fever (CCHF) cases that were detected during the SVD outbreak response.

**Methodology:**

Whole blood samples from VHF suspected cases were tested for Sudan virus (SUDV) by real-time reverse transcription–polymerase chain reaction (RT-PCR); and if negative, were tested for CCHF virus (CCHFV) by RT-PCR. CCHFV genomic sequences generated by metagenomic next generation sequencing were analyzed to ascertain strain relationships.

**Principal findings:**

Between September 2022 and January 2023, a total of 2,626 samples were submitted for VHF testing at UVRI. Overall, 13 CCHF cases (including 7 deaths; case fatality rate of 53.8%), aged 4 to 60 years, were identified from 10 districts, including several districts affected by the SVD outbreak. Four cases were identified within the Ebola Treatment Unit (ETU) at Mubende Hospital. Most CCHF cases were males engaged in livestock farming or had exposure to wildlife (n = 8; 61.5%). Among confirmed cases, the most common clinical symptoms were hemorrhage (n = 12; 92.3%), fever (n = 11; 84.6%), anorexia (n = 10; 76.9%), fatigue (n = 9; 69.2%), abdominal pain (n = 9; 69.2%) and vomiting (n = 9; 69.2%). Sequencing analysis showed that the majority of identified CCHFV strains belonged to the Africa II clade previously identified in Uganda. Two samples, however, were identified with greater similarity to a CCHFV strain that was last reported in Uganda in 1958, suggesting possible reemergence.

**Conclusions/Significance:**

Identifying CCHFV from individuals initially suspected to be infected with SUDV emphasizes the need for comprehensive VHF testing during filovirus outbreak responses in VHF endemic countries. Without expanded testing, CCHFV-infected patients would have posed a risk to health care workers and others while receiving treatment after a negative filovirus diagnosis, thereby complicating response dynamics. Additionally, CCHFV-infected cases could acquire an Ebola infection while in the ETU, and upon release because of a negative Ebola virus result, have the potential to spread these infections in the community.

## Author summary

Crimean-Congo hemorrhagic fever (CCHF) is a severe hemorrhagic disease whose symptoms mimic those of other viral hemorrhagic fevers (VHFs), significantly challenging its differential diagnosis. Herein, we report on CCHF cases that were detected during the nationwide response activities against an outbreak of Sudan virus (SUDV) in Uganda, from September 2022 to January 2023. Confirmed CCHF cases, mainly males presenting with a range of clinical signs and symptoms, were identified in districts with circulating SUDV, including 4 cases identified within an Ebola treatment center at Mubende hospital, thus complicating patient management and treatment. Our findings highlight the need for broad testing for VHFs and other high-consequence pathogens, even during known VHF outbreaks, in order to identify high risk pathogens with outbreak potential, especially in countries with a broad range of endemic zoonotic viral pathogens. This article also adds to the known molecular epidemiology of CCHF virus in Uganda.

## Introduction

Crimean-Congo hemorrhagic fever (CCHF) has emerged as a disease of significant global public importance since it was reported as a novel infection among Russian military personnel stationed in the Crimean Peninsula in 1944. The disease, which was later found to be indistinguishable to the one that affected a 13-year old boy in the northeastern parts of the Democratic Republic of the Congo (DRC) in 1956 [1], has since been found to be widely spread in most of Africa, Eastern Europe, the Middle East and Asia [2]. CCHF is currently listed by the World Health Organization (WHO) as a priority disease for research and development due to a range of factors including a high case fatality rate, range expansion into non-endemic areas, lack of approved medical countermeasures and its potential for use as a bioterrorism agent [3]. Following the International Health Regulations (2005), outbreaks of CCHF, wherever they may occur, are notifiable to WHO and other international public health management bodies, with a primary objective for immediate containment [4].

The disease is caused by CCHF virus (CCHFV), with ixodid ticks from the *Hyalomma* genus, acting as the principal vector and reservoir [1,5]. Structurally, CCHFV is an enveloped RNA virus that belongs to the order Bunyavirales, family *Nairoviridae* and genus *Orthonairovirus*. The virus contains an approximately 19kb long, negative sense genome consisting of small (S), medium (M), and large (L) segments. The segments exhibit 65–76% nucleotide identity and reassortment has been described in the S and M segments [6]. Newly emerging strains and mixed genotypes have been reported in endemic countries [7] and within tick vectors [8]; a phenomenon that is widely recognized as a potential challenge to future diagnostic, therapeutic and vaccine interventions [9].

The natural transmission cycle for CCHFV represents a typical zoonosis, with humans acquiring infection through tick bites, or when they come in direct contact with body fluids from infected animals or other humans [2]. Generally, human CCHF cases are sporadic, with most presenting as asymptomatic or with mild forms of disease that may go unreported [10]. However, individuals who develop viremia progressing into fulminant disease, usually present with an acute onset of malaise (fever, chills, headache, fatigue, myalgia, abdominal pain, diarrhea) and eventually a severe hemorrhagic fever, with increased risk for secondary transmissions to close contacts [1,5]. A central challenge, however, to the clinical management of CCHF, is the lack of specific signs and symptoms among suspected cases, hence it is frequently misdiagnosed [11,12]. Reported mortality rates among CCHF confirmed cases vary widely depending on geographical regions and outbreak size, but can reach 30% or higher among hospitalized cases [13–15]. Additionally, VHF outbreaks in general may result in social panic and other short- and long-term economic, healthcare and cultural disruptions [16].

In Uganda, human CCHF cases have been identified since 1958 [17]; the majority of cases have been found within the cattle corridor–a contiguous zone that stretches diagonally from the Southwestern to the Northeastern parts of the country which is characterized by nearly exclusive livestock farming for economic livelihood [13]. Recent studies have suggested that CCHFV is present in other areas of Uganda where no historical human cases have been reported [18,19]. There are limited reports of person-to-person transmission of CCHFV in Uganda [13,20], and perhaps in most of Africa [21], in contrast to what has been reported during a recent outbreak in Pakistan [22] and previously in other Eurasian countries [23,24]. From a recent study by Temur *et al* [25], there is limited information on CCHF incident cases in Africa that is also responsible for underestimating the burden of disease in the region.

Herein, we report the descriptive epidemiology of 13 CCHF cases detected in Uganda within a period of 5 months, from September 2022 to January 2023, in association with heightened surveillance due to an ongoing outbreak of Sudan virus (SUDV) in multiple districts. These data demonstrate the molecular relatedness of CCHFV in Uganda and documents the evolution of the virus over time. We also present a case for broad testing of other viral etiologies during confirmed VHF outbreaks, especially in areas like Uganda, where other endemic high-consequence pathogens occur.

## Materials and methods

### Ethical approvals and consent to participate

This investigation was conducted within the context of responding to a public health emergency involving an outbreak of Sudan virus disease (SVD), following a directive by the Ministry of Health (MoH) of Uganda. Therefore, this work being for diagnostic purposes only, and primarily aimed for public health actions and disease control, mandatory institutional review clearance and consent to participate by patients is waived off, in accordance with the recent MoH guidelines [26]. This work was also reviewed by the US Centers for Disease Control and Prevention and found to be consistent with applicable federal laws and policies such as 45 C.F.R. 46.104. Nevertheless, all patients were verbally informed and agreed to a presumptive clinical suspicion of a viral hemorrhage fever at triage, and all diagnostic results (including the CCHF results reported here) were relayed back to them as soon as they were available. And throughout this report, all personal identifiable information related to patients and samples is de-identified.

### A brief summary of the Sudan virus outbreak

On 20 September 2022, the Ugandan MoH announced an outbreak of SVD in Mubende district. The virus was detected by the VHF Diagnostics Laboratory at Uganda Virus Research Institute (UVRI), Entebbe, Uganda, in a whole blood sample collected from a febrile 26 year-old male from Madudu village. The outbreak spread to 8 additional districts, mainly in central Uganda, before it was declared over on 11 January 2023. A total count of 142 confirmed and 22 probable cases were recorded. Epidemiological and other response-related activities associated with this SVD outbreak have been published elsewhere [27,28].

### Sample collection, RT-PCR diagnostic testing and high throughput sequencing

As a part of the SVD outbreak response, whole blood (EDTA) samples were collected from all VHF suspected cases throughout the country, for confirmation of SVD acute infection using a real-time reverse transcription PCR (RT-PCR) assay, as described elsewhere [29]. A suspected VHF case was defined as any individual with sudden onset of fever (≤38.5°C), followed by a variety of symptoms such as headache, fatigue, anorexia, abdominal pain, vomiting and/or hemorrhage, and with no alternative diagnosis (including but not limited to malaria, typhoid). As has been the practice during previous outbreak investigations of Ebola disease (EBOD) in Uganda [30,31], all SUDV-negative samples were also investigated for the presence of other viral infections such as other Ebolavirus species, Marburg, CCHFV and Rift Valley fever virus (RVFV). Briefly, once samples were submitted to the VHF Diagnostics laboratory at UVRI (primarily through the established national sample transportation network [32]), RNA was extracted using MagMAX RNA isolation kits (Thermo Fisher Scientific, Vilnius, Lithuania) according to the manufacturer’s procedures. CCHFV-specific RT-PCR was performed using the Superscript III Platinum One-Step qRT-PCR Kit (Life Technologies Corporation, CA, USA) and in-house custom primers (CCHFV-S-4-F: CAA AGA AAC ACG TGC CGC TT; CCHFV-S-79-R: ATT CAC CTC GAT TTT GTT TTC CAT) and probe (CCHFV-S-24-Prb: 5’ 6-FAM-AC GCC CAC A/ZEN quencher/G TGT TCT CTT GAG TGT TAG CA-3’) that target the CCHFV S-segment, as previously published [33]. All RT-PCR CCHFV positive samples were sequenced using previously described methods [13].

### Bioinformatics and phylogenetics

CCHFV genomes were *de novo* assembled using SPAdes (-k auto, v3.14.0) [34] and contigs were blasted to identify the most closely related reference sequences [13,35]. Genomes were iteratively assembled twice (using Geneious assembler) to incorporate variants relative to the initial reference sequence. Consensus genomes were generated using Geneious (Assign Quality = Total, Coverage > 2) [36]. Evolutionary history was inferred using all available full-length CCHFV genomes from GenBank using RAxML (-m GTRGAMMA -p $RANDOM -f a -x $RANDOM -N 1000) with bootstrap support provided by 1,000 iterations [37]. CCHFV genomes were deposited to the GenBank and are available with Accession Numbers OR522725-OR522752. To obtain a more comprehensive and updated picture of the CCHFV molecular epidemiology in Uganda, genomic data from two earlier CCHF cases (2022000484 and 2022001211 as shown in Fig 3) was included. These additional cases had been identified from the ongoing CCHF surveillance efforts in March and July 2022, respectively.

### Data analysis

Patient data including demographics (age, sex, and occupation), presenting signs and symptoms, location of districts where the case was detected, exposure factors and illness outcome, was abstracted from case investigation forms and summarized into MS Excel (Microsoft Corporation, Redmond, WA, USA). A simple descriptive analysis was performed by way of determining frequency and percent distributions for categorical variables, while means and/or medians were computed for continuous data.

## Results

### Identification of CCHF cases concurrent with the SUDV outbreak in Mubende

**Fig 1:**
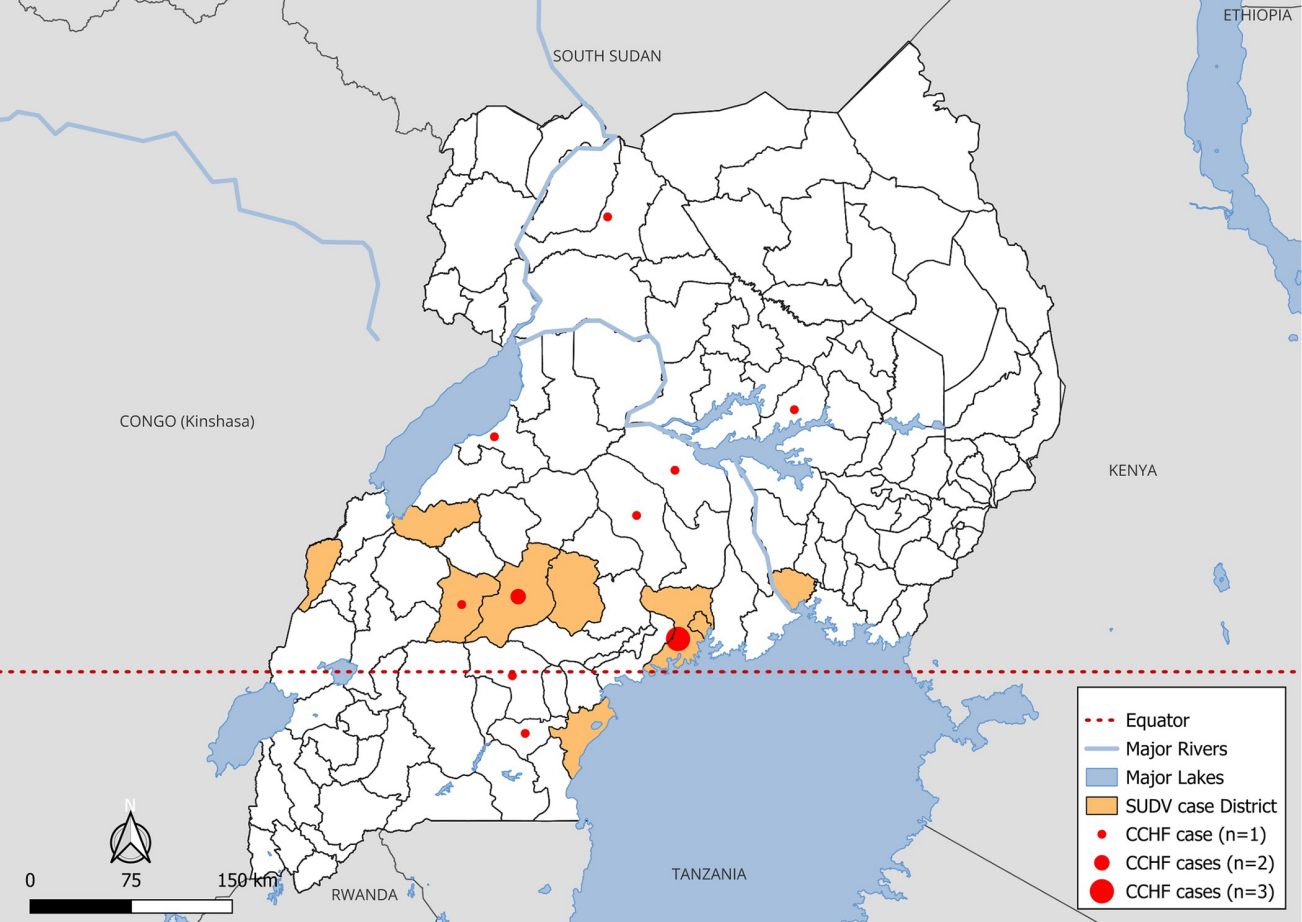
Map of Uganda showing the geographic location of Crimean-Congo hemorrhagic fever cases in relation to Sudan virus disease outbreak, September 2022- January 2023. This map was created using QGIS, v 3.22.5, a free and open-source geographic information system available at: https://qgis.org/project/overview/. The Uganda map base layers were accessed from Humanitarian Data exchange v.1.83.2 (Uganda administrative boundaries - UGANDA BOUNDARIES SHAPEFILES AS OF 17 08 2018.zip - Humanitarian Data Exchange (humdata.org).

**Fig 2:**
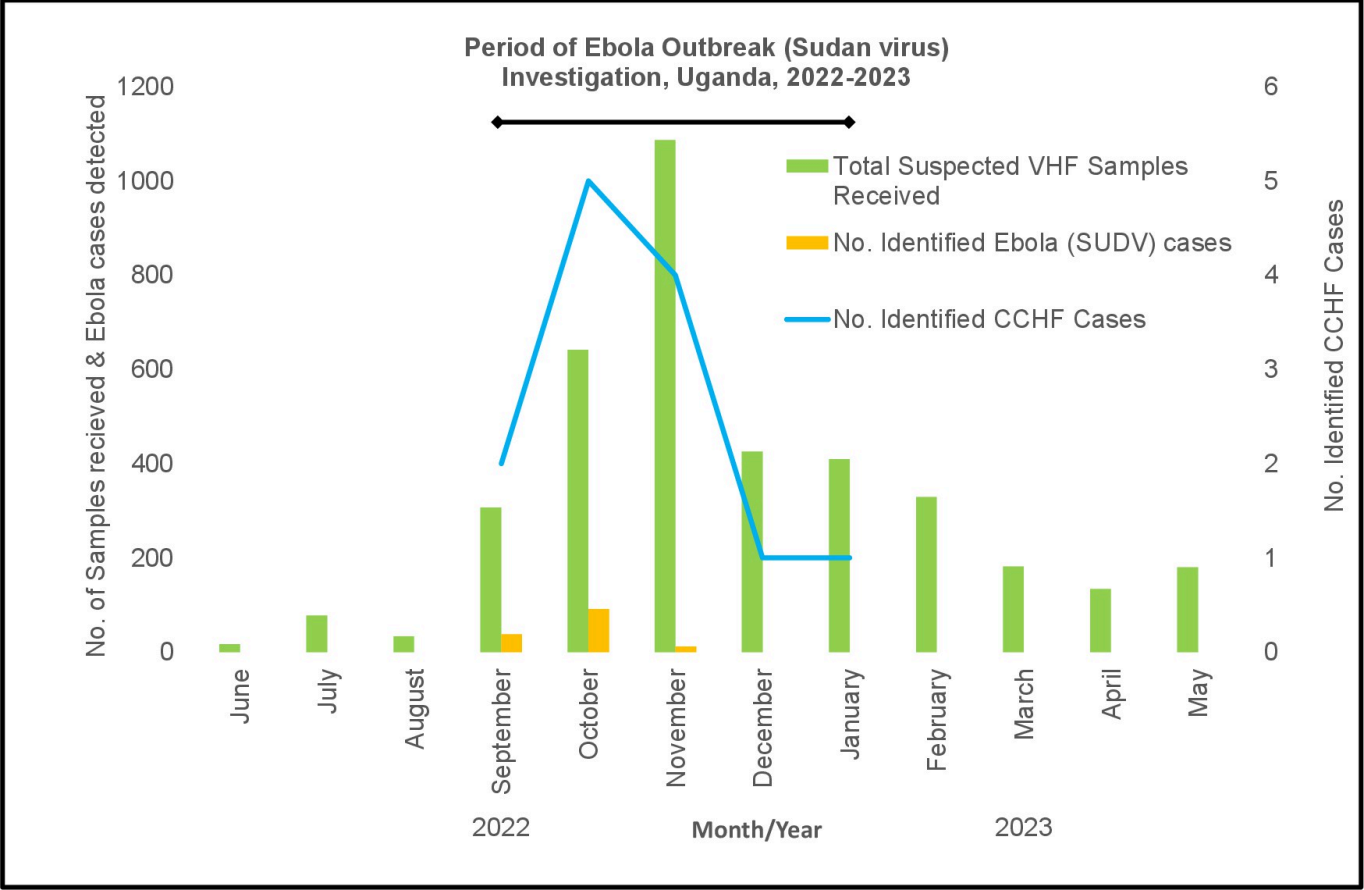
Trends of viral hemorrhagic fever suspected samples received and cases of Ebola disease (SUDV) and Crimean-Congo hemorrhagic fever identified between June 2022 to May 2023 at Uganda Virus Research Institute, Entebbe, Uganda.

During the SUDV Mubende outbreak in Uganda, a total of 2,626 specimens were received at UVRI for suspect VHF diagnostics. Specimens were first tested for SUDV, followed by testing for other filoviruses (Zaire, Bundibugyo and Marburg), CCHFV and RVFV. From September 2022 to January 2023, a total of 142 confirmed and 22 probable SUDV cases were identified [27]. During this time, 13 CCHFV-positive cases were also identified in 10 districts, including in 4 individuals from the districts of Mubende (n = 2), Kyegegwa (n = 1) and Hoima (n = 1) that were initially suspected for SVD and their specimens were collected in the SUDV ETU (Figs 1 and 2). The remaining CCHF-positive specimens were submitted from surveillance hospitals and health centers located around Uganda. There was a more than ten-fold increase in suspect specimens sent to UVRI for VHF diagnostics during the SVD outbreak (Fig 2).

The demographic and clinical presentations of CCHF-positive cases have been summarized in Table 1. Cases had an average age of 35.4 years (Range: 4–60 years). And similar to previous CCHF cases that have been reported in Uganda [13,20], a majority of cases we report here were males (61.5%), above 20 years of age (61.5%), living within the cattle corridor zone (61.5%) and practicing some agricultural or veterinary activities, and/or with direct contact to wildlife (53.8%). However, case fatality rate (CFR) was 53.8% which was higher than the 30–35% CFR observed in the previous investigations.

**Table 1:** Baseline Characteristics of Crimean-Congo Hemorrhagic Fever Cases in Uganda, September 2022 –January 2023 (n = 13).

Description		Number	%
Gender	Male	8	61.5
	Female	5	38.5
Age (Yrs.)	Mean, Range	35.4, 4–60	
Age Distribution (Yrs.)	0–10	1	7.7
	11–20	2	15.4
	21–30	0	0.0
	31–40	2	15.4
	41–50	4	30.8
	50+	2	15.4
	Unknown	2	15.4
Occupation	Farmer	5	38.5
	Animal Keeper/Hunter	2	15.4
	Student/Child	2	15.4
	Laborer	1	7.7
	Unknown	3	23.1
Clinical Signs and Symptoms	Bleeding	12	92.3
	Fever	11	84.6
	Anorexia	10	76.9
	Fatigue	9	69.2
	Abdominal pain	9	69.2
	vomiting	9	69.2
	Headache	7	53.8
	Diarrhea	5	38.5
	Chest pain	5	38.5
	Joint pain	5	38.5
	Difficult breathing	5	38.5
	Muscle pain	4	30.8
	Cough	4	30.8
	Jaundice	4	30.8
	Confused	3	23.1
	Conjunctivitis	2	15.4
	Difficult swallowing	2	15.4
	Hiccups	2	15.4
	Sore throat	1	7.7
Clinical Outcome	Survived	6	46.2
	Died	7	53.8

### Inferred relatedness of recent CCHFV-positive specimens

**Fig 3:**
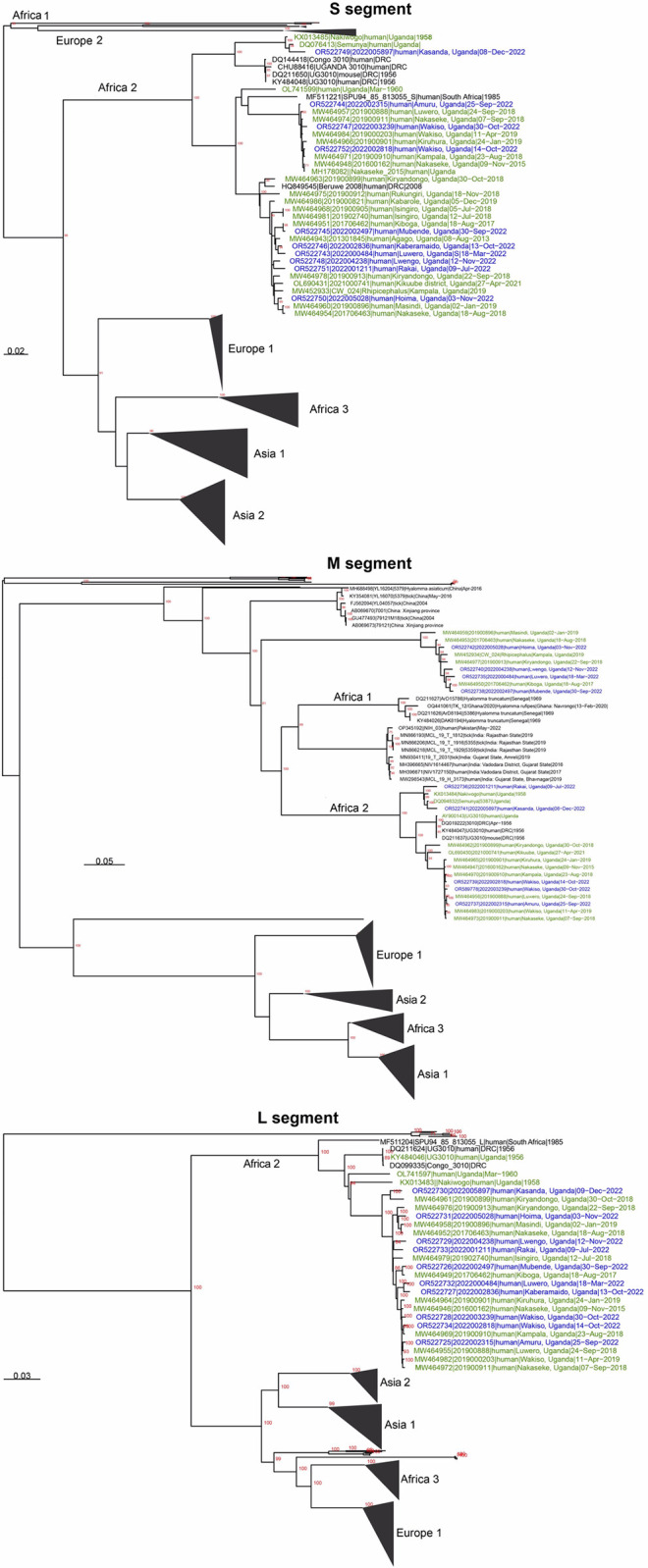
Inferred evolutionary relationships for all available full length CCHFV genomes, including recent and historic CCHFV genomes from Uganda. Historic (1956–2019) genomes are highlighted green, and recent (March-December 2022) genomes are highlighted blue. The S-segment phylogenetic tree contains two recent Ugandan-specific clades, in addition to a third clade containing two sequences from the 1950s and one from 2022. The M-segment phylogenetic tree includes Ugandan sequences split onto two clades. Two recent sequences (2022001211 and 2022005897) cluster with historic Ugandan sequences from the 1950s. While the L-segment phylogenetic tree includes a Uganda/DRC specific Africa 2 clade.

Coding complete genomic sequences were obtained for 10 L segments, 9 M segments, and 10 S segments out of the 13 CCHF positive samples that were collected during the study period. Phylogenetically, a majority of the new CCHFV-positive cases clustered with historical Ugandan sequences (1956, 2013–2019) on the L-, M-, and S-segment Africa II clade (Fig 3). These new sequences were also evenly distributed on the two CCHFV M clades previously identified in Uganda. With the addition of the new sequences from this investigation as shown in Fig 3, there is now strong bootstrap support for the Uganda M and S clades separating into well-supported two clades. Of particular note, two M segments (2022001211 and 2022005897) were most closely related to sequences collected in 1958. Similarly, the 2022005897 S-segment was also most closely related to the 1958 sequences, whereas the 2022001211 S-segment was most closely related to an S-segment from 2022, strongly suggesting that the 2022001211 M-segment represents a reassortment event.

## Discussion

In this study, a total of 13 CCHF cases were identified in Uganda between September 2022 and January 2023 that corresponds to a period within which there was also enhanced surveillance for EBOD throughout the country. The higher number of CCHF cases detected within a short period (5 months) in Uganda, is reminiscent of what was observed during a similar enhanced EBOD surveillance period in 2018–19, when 14 CCHF cases were detected from multiple areas of Uganda [20]. This period occurred during an ongoing EBOD outbreak in neighboring eastern DRC, which led to the activation of enhanced disease surveillance activities throughout Uganda [30]. The sudden increment of case counts in association to increased surveillance activities, in these two scenarios, allude to the fact that the CCHF burden in Uganda, may be bigger than has been determined through routine surveillance and that several human CCHF cases do occur, but go undetected. Indeed, recent non-outbreak associated studies in the country, have shown high circulation levels of CCHFV in domestic animals and some tick populations [18,19,38], in variance to the small, or usually single and sporadic cases that are reported during several outbreak situations in the same areas [13,31,39–41]. Whereas routine surveillance for CCHF in Uganda has been implemented over the last 10 years [13,31], it is still not adequately spread out in the countryside [42], and it appears there is generally limited public and professional awareness about the disease [43]. The underestimation of the CCHF burden on the African continent, generally, was recently investigated by Temur *et al* [25], and they reported poor case detection and the overall lack of effective VHF surveillance activities as important factors for this anomaly. It is also worth noting that a majority of the cases we report here had fulminant disease as a trigger for suspecting a VHF infection. Therefore, given reports from elsewhere that subclinical CCHF infections are the majority of human cases [10,44], it is prudent to believe that additional cases are likely to have been missed by the Ugandan health system during non-enhanced surveillance periods, especially those who present with mild illness. As we have recently suggested [45], improving the index of suspicion by clinicians for potential CCHF cases, as well as public awareness about VHFs in general, are key factors for reducing the burden of CCHF in the immediate future for Uganda. However, these will need to be supplemented with effective tick control practices and implementing a broader research agenda on the endemic characteristics of CCHF including the one health approach.

Viral sequences were generated and whole genomes analyzed for a majority of the CCHF cases identified during the SVD outbreak; an additional 2 sequences from samples that were collected a few months before the outbreak were also included for completeness. This investigation is an addition to similar molecular investigations for CCHFV that our team and others have carried out in the recent past [13,39–41]. Altogether, this makes Uganda one of the African countries with the highest amount of publicly-available CCHF genetic data. Broadly, the CCHF virus in Uganda remains in the Africa II (for L- and S-segments) and Africa I and II (M-segment) clades as previously observed [13]. With the additional sequence data from this investigation, there is now strong bootstrap support for the Ugandan S clade separating into two distinct clades, similar to the two M-segment clades previously observed in Uganda. Perhaps in agreement with previous findings that CCHFV’s evolutionary change is relatively low over long periods of time within geographical localities [46,47], the S-segment from one case (2022005897) and M-segments from two cases (2022005897 and 2022001211), exhibited similarity to a 1950s CCHFV strain. It is particularly noteworthy that the 2022005897 and 2022001211 strains also contain segments with similarity to recent CCHFV strains, perhaps demonstrating evidence of reassortment. These data may suggest that a stable 1950s clade has continued to circulate in the environment at low and undetected levels over the past 65 years, with perhaps infrequent incidents of spillover to humans, thus making the identification of these two samples a mere chance of random encounter. However, it could also indicate that the 1950s strain may be re-emerging, after the human population in the area has become immunologically competent against the prevalent strains, and inversely to historical ones. And the reassortment with historic strains could represent a mechanism that introduces glycoprotein and nucleoprotein variation. To date, little is known about the naturally acquired immunity against CCHF in humans including its long-term durability and cross protection against different lineages [48].

We were unable to perform field investigations to obtain detailed information on exposure risks, vector populations and other associated environmental factors responsible for a high number of CCHF cases within a short investigation period. This work was done by Uganda MoH field teams, to whom the primary focus was on identifying Ebola virus-infected cases. Nevertheless, our data in this report emphasizes the need to apply comprehensive testing during outbreak investigations, including beyond the etiologic agent of the outbreak. This is important for countries such as Uganda, where multiple disease agents co-circulate, many with indistinguishable clinical manifestations [49]. Indeed, the CCHF cases reported in this manuscript were not clinically different from those that had EBOD (Table 1)[50]. In Uganda, the current approach in public health emergency responses, narrowly focuses on the management of the involved pathogen; which limits the ability to use the available funding for enhanced diagnostics and logistics for the detection of other pathogens with similar clinical symptoms. Yet as presented here, other pathogens can cause undetected simultaneous outbreaks. Broad diagnostic surveillance is key to detecting clinically similar, but unrelated, outbreaks. Interestingly, anecdotal data from some CCHF endemic countries such as Turkey and Pakistan, now show CCHF as a coinfectant in some cases of COVID-19 and other endemic infections [51–53], potentially impacting their pathophysiology.

These data call for enhancement of the current surveillance system for VHFs in Uganda, including increasing public awareness and a higher index of clinical suspicion for CCHF infection by the health infrastructure, as it appears that many CCHF cases remain undetected. The increased vigilance due to the ongoing EBOD outbreak identified many CCHF cases within a short period that could have been missed during routine surveillance. By extrapolation, these data suggest a high CCHF burden in Uganda, and that the country could benefit from medical countermeasures, such as vaccines and acaracides, which could reduce disease prevalence [44,54]. We therefore recommend the implementation of improved surveillance through serosurveys and other population-based surveillance methods so that the true burden of CCHF infection in Uganda can be accurately determined.
